# Usefulness of the Upright Posture Test in the Diagnosis of Primary Aldosteronism

**DOI:** 10.1210/jendso/bvae155

**Published:** 2024-09-04

**Authors:** Nada Younes, Matthieu St-Jean, Marie-Josée Desrochers, Eric Therasse, Mathieu Latour, Isabelle Bourdeau, André Lacroix

**Affiliations:** Division of Endocrinology, Department of Medicine and Research Center, Centre hospitalier de l’Université de Montréal, Montréal, Québec, Canada, H2X 0A9; Division of Endocrinology, Department of Medicine, Centre hospitalier de l’Université de Sherbrooke, Sherbrooke, Québec, Canada, J1H 5H3; Division of Endocrinology, Department of Medicine and Research Center, Centre hospitalier de l’Université de Montréal, Montréal, Québec, Canada, H2X 0A9; Department of Radiology, Centre de Recherche du Centre hospitalier de l’Université de Montréal, Université de Montréal, Québec, Canada, H2X 0A9; Department of Pathology and Cellular Biology, Centre hospitalier de l’Université de Montréal, Montréal, Québec, Canada, H2X 0A9; Division of Endocrinology, Department of Medicine and Research Center, Centre hospitalier de l’Université de Montréal, Montréal, Québec, Canada, H2X 0A9; Division of Endocrinology, Department of Medicine and Research Center, Centre hospitalier de l’Université de Montréal, Montréal, Québec, Canada, H2X 0A9

**Keywords:** primary aldosteronism, diagnosis, confirmation test, upright posture, saline tests

## Abstract

**Objective:**

To assess the usefulness of the upright posture stimulation test (UPT) in the confirmation of primary aldosteronism (PA) in patients in whom saline tests (ST) were inconclusive.

**Methods:**

One hundred eighty-seven adult patients with possible PA were retrospectively included and compared to 25 control subjects. Blood samples were obtained after a 1-hour supine posture and during 2 hours of ambulation. An increase in plasma aldosterone concentration (PAC) ≥ 50% with a suppressed renin (≤10.1 ng/L; ≤1 ng/mL/hour) and a cortisol increase ≤50% were considered abnormal.

**Results:**

PA patients had higher basal PAC and lower basal direct renin concentration (DRC) (*P* < .0001) and a higher maximal PAC (*P* = .0025) and lower maximal DRC (DRC_max_) (*P* < .0001) during UPT compared to controls. PA was confirmed in 145 patients (77.5%), based on either oral/IV ST or UPT. DRC_max_ ≤12 ng/L during UPT was a predictor of PA (receiver operating characteristic curve sensitivity 93.8%, specificity 88%), and 95.6% of PA patients increased PAC ≥50% on UPT (median 222.2%), while renin remained suppressed. All 41 PA patients with false-negative IV ST (PAC < 162 pmol/L) and 88.9% with borderline response (162-240 pmol/L) had a DRC_max_ ≤12, while, respectively, 97.6% and 100% increased aldosterone by ≥50%. Similar responses to UPT were found in lateralized (28/63) and bilateral PA source (35/63). PA diagnosis increased from 23.6% to 88.8% using UPT results instead of IV ST and were confirmed at pathology and clinical outcome after adrenalectomy (n = 22).

**Conclusion:**

UPT can be useful to confirm PA, particularly in patients with suspected false-negative ST.

Primary aldosteronism (PA) is a spectrum of diseases caused by excess renin-independent secretion of aldosterone; its manifestations range from normotension to severe hypertension, with or without hypokalemia, and it results in increased cardiovascular morbidity and mortality [1]. PA is clearly underdiagnosed in the general population, and prevalence varies according to the studied population [2]. It was diagnosed in 5.9% of patients with hypertension in the primary care setting [3] and more than 20% of patients with resistant hypertension [4-6]. However, 11.3% of normotensive patients had a high urinary aldosterone with a suppressed plasma renin activity (PRA) following a high sodium balance [4], further corroborating that PA is underrecognized, especially in its early normotensive stage. Screening for PA is based on the measurement of the aldosterone to renin ratio (ARR), preferably in the morning, after at least 2 hours of ambulation, and in a seated position to increase sensitivity [1, 7]. However, ARR cut-off values are highly variable, depending on the assays and units that are used for aldosterone and renin measurements [1, 8-10]. Because of the intraindividual variability of aldosterone concentrations, there is a fundamental shift toward relying on suppressed renin levels for PA screening and more than 1 ARR measurement rather than a single ARR value [10-13]. A positive screening for PA is usually followed by confirmatory testing. In cases of highly probable PA, suspected on the basis of: hypokalemia, an undetectable renin, and plasma aldosterone concentrations (PAC) > 550 pmol/L (>20 ng/dL), confirmatory testing can be bypassed according to the Endocrine Society guidelines, the French Society of Endocrinology, and the Japanese Endocrine Society [8, 14, 15]. The most widely used tests in clinical practice are the seated intravenous (IV) or oral saline loading tests (ST), the captopril challenge test, and, less frequently, the fludrocortisone suppression test [7, 8, 14]. In contrast, the Japanese Endocrine Society recommends confirmatory testing using either IV/oral ST, the captopril challenge test, or the furosemide-upright posture test [15]. The latter, which is almost exclusively used in Japan, consists of maintaining a supine posture for 30 minutes, followed by 40 mg of IV furosemide and keeping the patient in an upright posture for 2 hours afterwards [16-19]. The rationale behind this test is that in PA, renin is suppressed by the dysregulated overproduction of aldosterone and cannot be further stimulated despite a combination of 2 potent stimulators of the renin-angiotensin system: the administration of furosemide, which increases sodium urinary excretion and reduces blood volume and the upright posture [18, 19]. The test confirms PA when the PRA remains below 2 ng/mL/hour [or direct renin concentration (DRC) < 8 pg/mL] at 1- and 2-hours post furosemide injection [15, 16, 20], with a sensitivity of 93% [20]. A recently modified version of this test is the oral furosemide test using 80 mg of oral furosemide, followed by serial DRC measurements at 2 and 3 hours (posture not clearly indicated) [21]; a DRC below 7.6 μU/mL at 2 hours was 90% accurate for PA diagnosis [21].

Three to 5 decades ago, the upright posture stimulation test (UPT) was performed hoping to distinguish etiologies of PA, mainly aldosterone-producing adenoma (APA) from bilateral adrenal hyperplasia (BAH), but was later abandoned because of significant overlap in responses [22-26]. It was more recently reassessed for the same purpose, and results were contradictory. One study found it correctly detects lateralized PA when PAC decreases by at least 28% 4 hours following upright posture stimulation, with a sensitivity of 100% [27]. In contrast, others found the test to be of no clinical usefulness because of its inability to correctly discriminate between the 2 etiologies [18, 20, 28]. In both APA and BAH, aldosterone increases in response to posture stimulation while renin remains in a suppressed state [20, 28, 29]. Our group has previously shown that renin-independent aldosterone production is not completely autonomous as aldosterone secretion is regulated by various stimuli which activate a diversity of aberrant G-protein coupled receptors (GPCR) expressed in APA and BAH [29-31]. Aldosterone increased in response to several stimuli equally in patients with APA and BAH, such as the mixed meal test, ACTH 1-24, gonadotropin-releasing hormone, and the UPT [29]. The latter produced a positive response (ie, an aldosterone increase ≥50% with ACTH and renin below the lower limit of normal) in 83% of BAH and 71% of APA, which can potentially be explained by the activation of aberrant ß-adrenergic, vasopressin, or yet to be identified receptors in adrenal tissues of patients with PA [29, 32]. This implied that the UPT could possibly be used as a confirmatory test for a great proportion of PA, regardless of its etiology. To further elucidate this hypothesis, we retrospectively analyzed all patients diagnosed with PA who had undergone an UPT during their investigation for PA in our 2 referral university centers.

## Materials and Methods

### Patient Selection Criteria

We retrospectively analyzed the medical records of 187 adult patients with arterial hypertension who underwent a UPT as a second-line confirmation test during evaluation for possible PA at Centre hospitalier de l’Université de Montreal (CHUM) and Centre hospitalier de l’Université de Sherbrooke between January 2011 and December 2021. We also included 25 control subjects: 10 normotensive healthy subjects and 15 normotensive patients investigated for unilateral or bilateral adrenal nodules and mild cortisol production [cortisol >50 nmol/L post 1 mg dexamethasone suppression test (DST) without clinical signs of Cushing's syndrome], with normal ARR and normal aldosterone and renin response to UPT. Diagnosis of PA was made according to the diagnostic criteria recommended by the Endocrine Society guidelines for the diagnosis and management of PA [8]. Demographic characteristics including age at the time of testing, sex, duration of hypertension, number of antihypertensive medications, blood pressure, and the need for potassium supplements were recorded. The highest ARR recorded in the medical file was also documented, along with adrenal imaging abnormalities and the results of the 1 mg overnight DST, the IV or oral ST results, adrenal vein sampling (AVS) results, and postadrenalectomy histopathology reports, whenever present.

PA was confirmed with either a positive seated IV ST when PAC was ≥162 pmol/L at 4 hours following a saline infusion [33] or a 3-day oral ST when urinary aldosterone was >38 nmol/day with a urinary sodium above 200 mmol/day [8, 29] or an abnormal response to the UPT (discussed later with more details). Before 2012, the IV ST was done in a supine posture and the cut-off of 240 pmol/L was used to determine an abnormal response [8]. Thus, IV ST results were subdivided into 3 categories: a clearly positive result when PAC was ≥240 pmol/L, a borderline result when PAC was between 162 and 240 pmol/L, and a normal response when PAC was <162 pmol/L. Both positive and borderline results were used to confirm PA.

**Figure 1.**
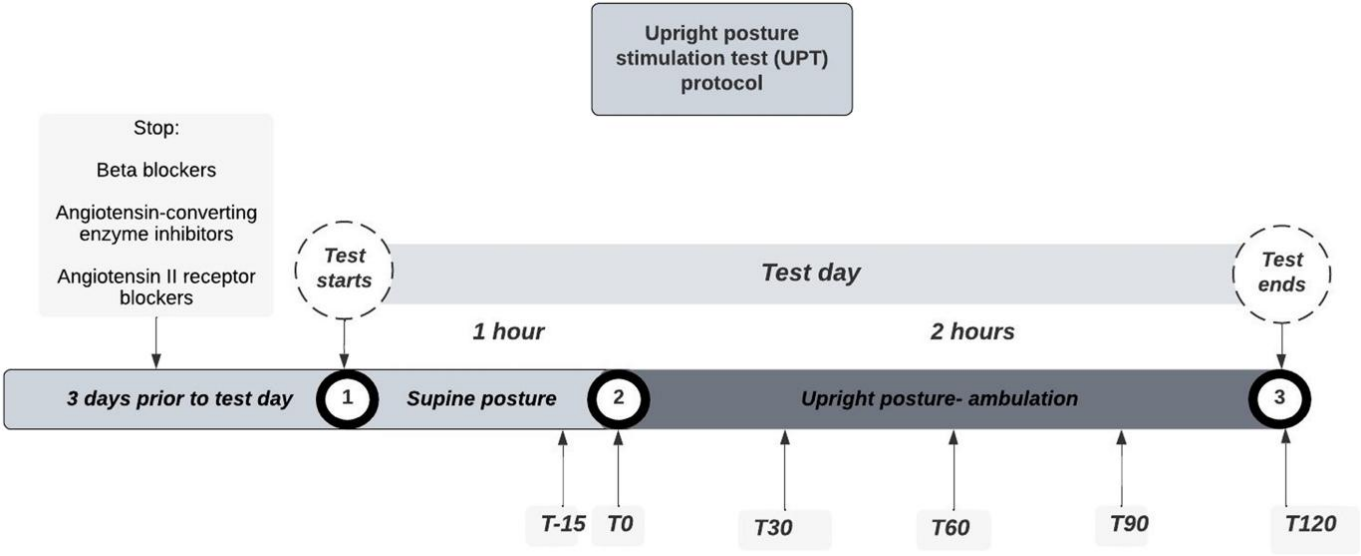
A detailed protocol of the upright posture stimulation test used in our 2 university centers. Blood samples are drawn at T-15 minutes and T0 minutes before ambulation; then at T30, T60, T90 and T120 minutes after the start of ambulation for measurement of plasma aldosterone concentration, direct renin concentration, or plasma renin activity and serum cortisol concentration.

When results of saline tests were negative but clinical suspicion remained high in the presence of either severe refractory hypertension (ie, blood pressure ≥160/100 mmHg or on more than 3 antihypertensive medications, including a diuretic) or persistent hypokalemia and persistently suppressed renin despite diuretics, angiotensin-converting enzyme inhibitors, or angiotensin II receptor blockers angiotensin II receptor blockers, a UPT was performed; it is important to note that we did not perform a UPT in the majority of patients investigated for potential PA who had clearly abnormal intravenous or oral saline load. Patients were asked to stop beta-blockers and angiotensin-converting enzyme inhibitors or angiotensin II receptor blockers 3 days prior to testing day. Testing was done in an ambulatory setting as outpatients and patients were asked to remain in a supine position for 1 hour and then ambulate for 2 hours (Fig. 1). Blood samples for the measurement of PAC, DRC (after 2014), or PRA (before 2014) and serum cortisol (F) concentration were drawn before ambulation at T-15 and T0 minutes and then at 30 minutes intervals following ambulation for 2 hours (T30, T60, T90, T120). The hypothesis was that normal subjects respond to an upright posture by increasing both renin and aldosterone proportionally [34], while in PA, aldosterone could increase independently from renin, which remains low. We utilized, as previously detailed [29], the following criteria for an abnormal response to the UPT: an increase of ≥50% in PAC, accompanied by a DRC ≤10 ng/L or PRA ≤1 ng/mL/hour and an increase of ≤50% in cortisol (C). The latter criterion was added to exclude the ACTH-dependent rise in PAC in patients with PA [22, 29, 32, 35]. A small subset of patients (n = 63) also underwent AVS to evaluate surgical indication, regardless of imaging findings. AVS was considered successful when adrenal vein F to inferior vena cava F gradient was 2:1 basally and/or >5:1 post ACTH 250 mcg IV bolus. Basal lateralization ratio ≥ 2 or post-ACTH ≥4 was considered consistent with lateralized disease [36]. Biochemical and clinical outcomes after surgery were evaluated according to the PASO criteria [37].

The ethics committee at both institutions waived the requirement to obtain written consent from included patients because of the retrospective nature of this analysis and because of anonymization of recorded and published data. For the control subjects, the CHUM ethics committee approved the study protocol, and written informed consent was obtained to conduct the UPT.

### Hormonal Assays

PAC was measured with 3 different radioimmunoassays during the period of the study [coefficient of variation (CV) < 10%], the latest one being the DIAsource ImmunoAssays, S.A. (Louvain-la-Neuve, Belgium, DiaSource Cat# 5331235, RRID:AB_2916289). Some patients before 2014 had a measurement of PRA performed with a radioimmunoassay (GammaCoat Plasma Renin Activity, DiaSorin Inc., Stillwater, MN, USA, DiaSorin Cat# CA1533, RRID:AB_2736926), CV <9%. However, after 2014, all patients had DRC measurements using an immunoradiometric method (RENINE III GENERATION Cisbio Bioassays, Codolet, France, CISBIO Cat# RENINE, RRID:AB_3166173), CV <7.5%. A conversion factor of 1 ng/mL/hour = 10 ng/L from PRA to DRC was used to facilitate statistical analysis in our population, despite the reported poor correlation between both measurements for suppressed PRA (<1 ng/mL/hour) [8].

Serum F was measured using a competitive chemiluminescent immunoassay (Siemens Healthcare Diagnostics Inc, Tarrytown, NY, USA, Siemens Cat# 10994924, RRID:AB_2893154), CV <8%. CVs in our laboratory were obtained from cumulative statistical analysis of internal quality control programs including 3 different concentrations for each hormone.

### Histopathology on Resected Adrenal Tissues

Hematoxylin and eosin slides of the adrenal glands resected from patients with lateralized source of aldosterone were analyzed by an experienced pathologist (M.L.) to confirm the presence of adenomas, nodular hyperplasia, or BAH. CYP11B2 staining was performed on 3-mm thick sections of deparaffinized tissue, and antigens were retrieved. Slides were incubated with mouse monoclonal antibodies (Ventana BenchMark system) against CYP11B2 (anti-CYP11B2, clone 41-17B, dilution 1:100—Millipore Sigma: RRID: AB_2783793). External positive controls were performed. Staining was scored semiquantitatively: diffuse vs heterogeneous or focal for distribution and high vs low for intensity.

### Statistical Analysis

The baseline characteristics and UPT measurements of patients and control subjects are presented as median (interquartile range) or as counts and percentages. When a measurement was available for 2 groups, the comparison was done using the chi-squared test and Kruskal–Wallis test, depending on the data distribution. Similar descriptions and comparisons were provided for the subset of patients with a PA diagnosis. Receiver operating characteristic (ROC) analyses were performed separately for each of the UPT parameters (PAC, DRC, F, and ARR with maximum and increase from basal values) as a predictor and patients with a PA diagnosis vs control subjects as an outcome. The area under the curve (AUC) and 95% confidence interval (95% CI) were calculated [38]. To compare different predictors, mean and 95% CI were computed for the differences of AUC [39]. The cut point values with their respective sensitivity and specificity for selected ROC curves were calculated based on the point closest to (0,1) corner. Descriptive analysis was presented for each of the following groups: (1) UPT in PA patients with normal or false-negative IV ST, (2) UPT according to lateralization on AVS, and (3) UPT in non-PA patients. Missing values were present in basal variables only and were excluded from the descriptive statistics. Despite cut-off values for positive supine and seated IV ST being different, we considered the same cut-off values regardless of position during testing. We justify this by the fact that patients who underwent a supine IV ST had either a nonequivocal positive response ≥240 pmol/L or a nonequivocal normal response <162 pmol/L, and no borderline results (PAC between 162 and 240 pmol/L) were documented.

All tests were conducted at a 2-sided .05 level of significance. Statistical software SAS version 9.4 was used for all calculations (SAS Institute, Cary, NC, USA).

## Results

### Baseline Characteristics

**Figure 2.**
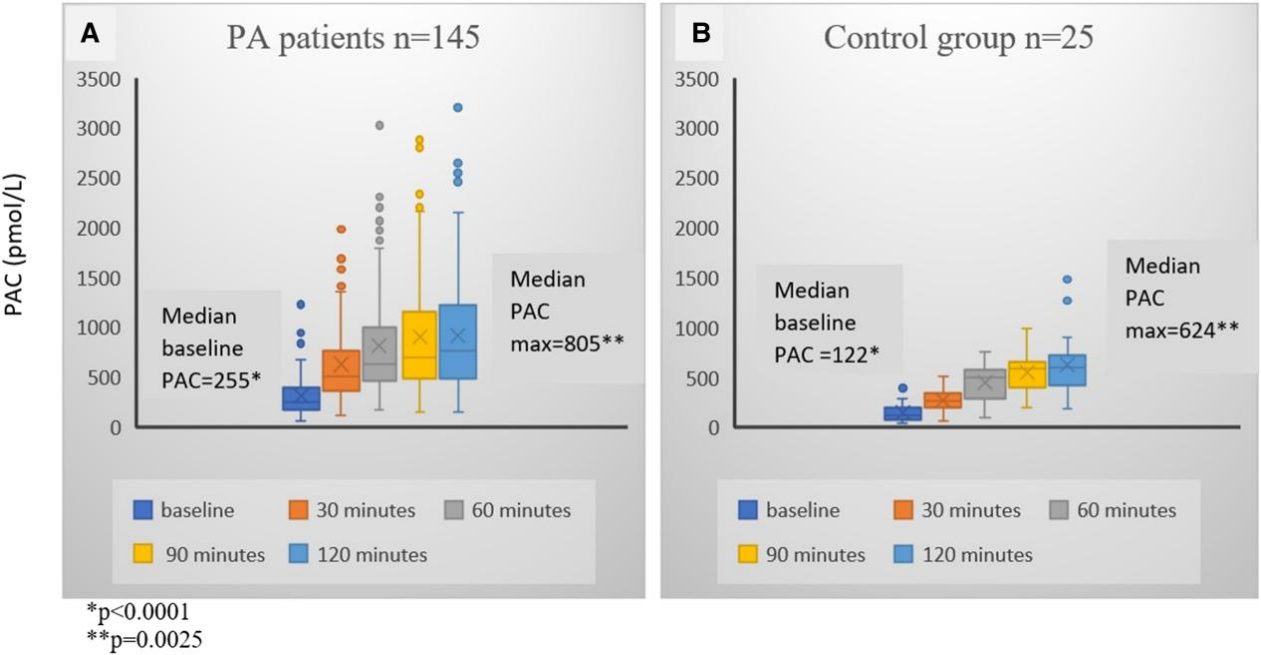
PAC (pmol/L) at baseline and during the upright posture stimulation test in patients with primary aldosteronism (PA patients) (A) and in control subjects (B). Abbreviations: PAC, plasma aldosterone concentrations.

**Figure 3.**
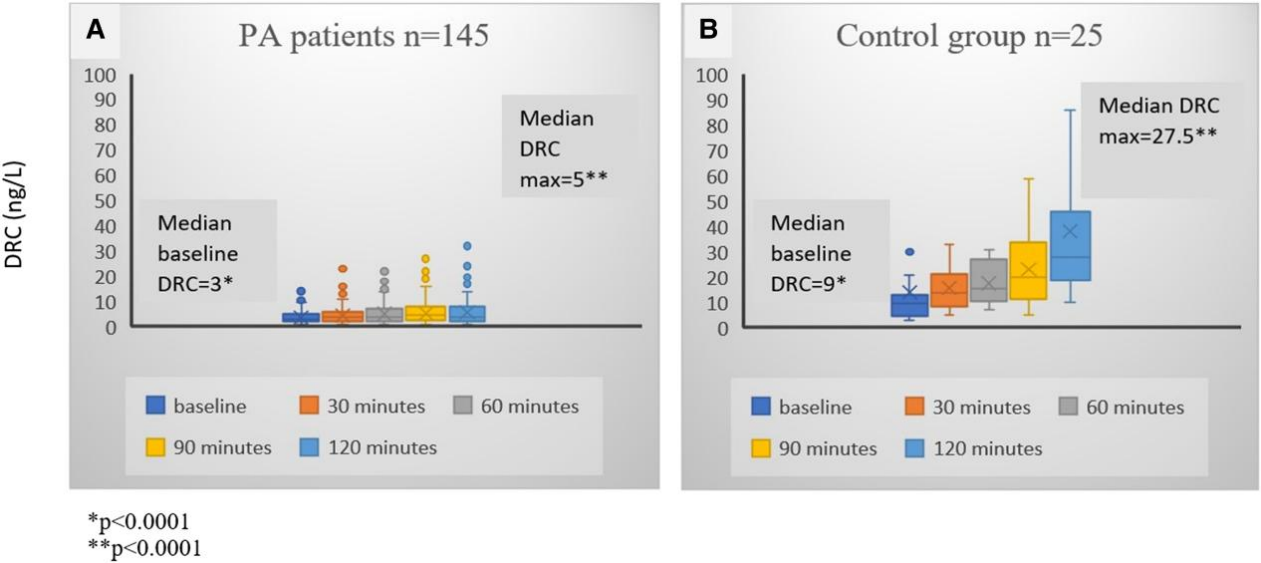
DRC (ng/L) at baseline and during the upright posture stimulation test in patients with primary aldosteronism (PA patients) (A) and in control subjects (B). Abbreviations: DRC, direct renin concentrations.

We performed the UPT in a total of 187 patients (173 patients from CHUM and 14 from Centre hospitalier de l’Université de Sherbrooke) in our study and 25 normotensive control subjects. Baseline characteristics for the total population are detailed in Table 1. One hundred forty-five patients (77.5%) were diagnosed with PA based on either IV (48 positive results of 89 tests performed), oral ST (27/53), or UPT (128/145, see details later) and were analyzed as a subgroup compared to controls. The remaining 42 patients without PA are detailed later in the section on non-PA patients. The baseline characteristics of the PA patients and that of controls are also presented in Table 1. There were no differences between the 2 groups regarding baseline characteristics available for comparison, except for lower systolic and diastolic blood pressure in the control group (*P* = .0002 and *P* = .0005, respectively). Additionally, 71.2% of patients with refractory hypertension (42 of 59 patients) had a positive PA diagnosis. As expected, when compared to controls, PA patients had higher basal PAC and lower basal DRC (*P* < .0001) (Figs. 2 and 3).

**Table 1.** Baseline characteristics of our study population (n = 187) of the patients diagnosed with PA (n = 145) and of the control subjects (n = 25)

Variable	Study population (n = 187)	PA patients (n = 145)	Control subjects (n = 25)	*P*-value*^a^*
Sex (%)				
Male	89 (47.6)	70 (48.3)	8 (32)	.13
Female	98 (52.4)	75 (51.7)	17 (68)	
Age (years)	55 (46, 63)	55 (47, 63)	52 (43, 61)	.27
Systolic BP (mmHg)	137.5 (126.5, 146.0)	139 (129, 148)	115.5 (110, 130)	.0002*^b^*
Diastolic BP (mmHg)	81.0 (75, 89.5)	83 (75, 90)	71.5 (63, 82)	.0005*^b^*
Potassium (mmol/L)	3.8 (3.5, 4.2)	3.7 (3.5, 4.2)		
Supplementary oral potassium (%)	60/185 (32.4)	50/144 (34.7)		
Supplementary oral potassium dose (mEq/day)	20 (20, 40)	20 (20, 40)		
Creatinine (µmol/L)	75.0 (63, 92)	74 (63, 91)		
ARR (pmol/L)/(ng/mL/h) (n = 39)	1550.3 (1064, 4260.0)	1730.2 (1290, 4500)		
ARR (pmol/L)/(ng/L) (*n* = 102)	141.7 (83, 216.8)	151.9 (96, 257.7)		
Nodules on adrenal imaging (%)				
Unilateral nodule	77/177 (43.5)	64/141 (45.4%)		
Bilateral nodules	23/177 (13)	16/141 (11.3)		
No nodules	77/177 (43.5)	61/141 (43.3)		
Size of largest nodule (n = 73) mm	14.0 (10, 19)	14 (10, 19)		
Number of antihypertensive medications (%)				
0	12 (6.4)	8 (5.5)		
1	54 (28.9)	44 (30.3)		
2	40 (21.4)	33 (22.8)		
3	26 (13.9)	19 (13.1)		
4	25 (13.4)	18 (12.4)		
5	25 (13.4)	19 (13.1)		
6	5 (2.7)	4 (2.8)		
At least 3 antihypertensive medications (%)	81 (43.3)	60 (41.4)		
Refractory hypertension (3 antihypertensive medications including one diuretic) (%)	59 (31.6)	42 (29)		
Abnormal 1mg-DST (>50 nmol/L) (%)	35/134 (26.1)	25/106 (23.6)		

Variables are presented as median (interquartile range) or n (%). Missing values were excluded from analysis.

Abbreviations: ARR, aldosterone to renin ratio; BP, blood pressure; DST, dexamethasone suppression test; PA, primary aldosteronism.

^*a*^Comparison was done between patients with PA and the control subjects.

^*b*^Statistically significant.

Regarding the response of PAC and DRC to upright posture stimulation, in the control group (n = 25), both PAC and DRC increased from baseline in response to upright posture, with a moderate correlation between percentage increases in PAC and DRC (Spearman correlation = 0.44). There was a significant difference in the response to the UPT seen with PA patients (n =145), compared to controls. PA patients achieved a higher maximal PAC (PAC_max_) in response to posture stimulation [805 (572, 1244) pmol/L in PA v/s 624 (500, 736) pmol/L in controls, *P* = .0025], whereas, the median DRC_max_ achieved was 5 (3, 8) ng/L and was significantly lower than that in the control group [27.5 (19, 46) ng/L; *P* < .0001] (Figs. 2 and 3 and Table 2).

**Table 2.** Comparison of response to the upright posture stimulation test between PA-confirmed patients and control subjects

	PA-confirmed patients (n = 145)	Healthy controls (n = 25)	
Variable	Median (IQR)	Median (IQR)	*P*-value
	(Min, Max)	(Min, Max)	
PAC basal (pmol/L)	255.0 (178.5, 388)	122 (79, 196)	**<**.**0001**
	(66, 1232.5)	(40, 422.5)	
PAC max (pmol/L)	805 (572, 1244)	624 (500, 736)	.**0025**
	(209, 3207)	(232, 1483)	
DRC basal (ng/L)	3 (2, 5)	9 (4.5, 13)	**<**.**0001**
	(1, 14)	(1.3, 110)	
DRC max (ng/L)	5 (3, 8)	27.5 (19, 46)	**<**.**0001**
	(1, 32)	(9.4, 172)	
C basal (nmol/L)	235 (169, 306.5)	220 (161.5, 323)	.63
	(36, 660)	(80, 613)	
C max (nmol/L)	306 (239, 393)	260 (198, 388)	.17
	(45, 789)	(106, 732)	
ARR basal (pmol/L/ng/L)	83.8 (51.5, 147.3)	16.3 (10.6, 26.3)	**<**.**0001**
	(17.5, 439)	(0.4, 121)	
ARR max (pmol/L/ng/L)	206.5 (122.7, 352.5)	23.7 (14, 39)	**<**.**0001**
	(34.9, 1141)	(6.7, 190)	
PAC increase (%)	223.5 (148.5, 312.1)	382.5 (210.6, 631.6)	.**0070**
	(24.3, 1162)	(38.9, 1337.5)	
DRC increase (%)	50 (14.3, 100)	237.5 (122.2, 490)	**<**.**0001**
	(−33.3, 500)	(−21.8, 766.7)	
C increase (%)	21.3 (−1.6, 77.8)	19.4 (−2.7, 57.1)	.28
	(−33.8, 628.6)	(−31.8, 130.7)	
ARR increase (%)	141.9 (65.4, 241.8)	72.3 (12.7, 123.7)	.**0044**
	(−32.8, 661.3)	(−48, 1738.7)	

Bold *P*-value ≤.05 = statistically significant.

Abbreviations: ARR, aldosterone to renin ratio; C, cortisol concentration; DRC, direct renin concentration; IQR, interquartile range; Min, minimal value; Max, maximal value; PA, primary aldosteronism; PAC, plasma aldosterone concentration.

**Figure 4.**
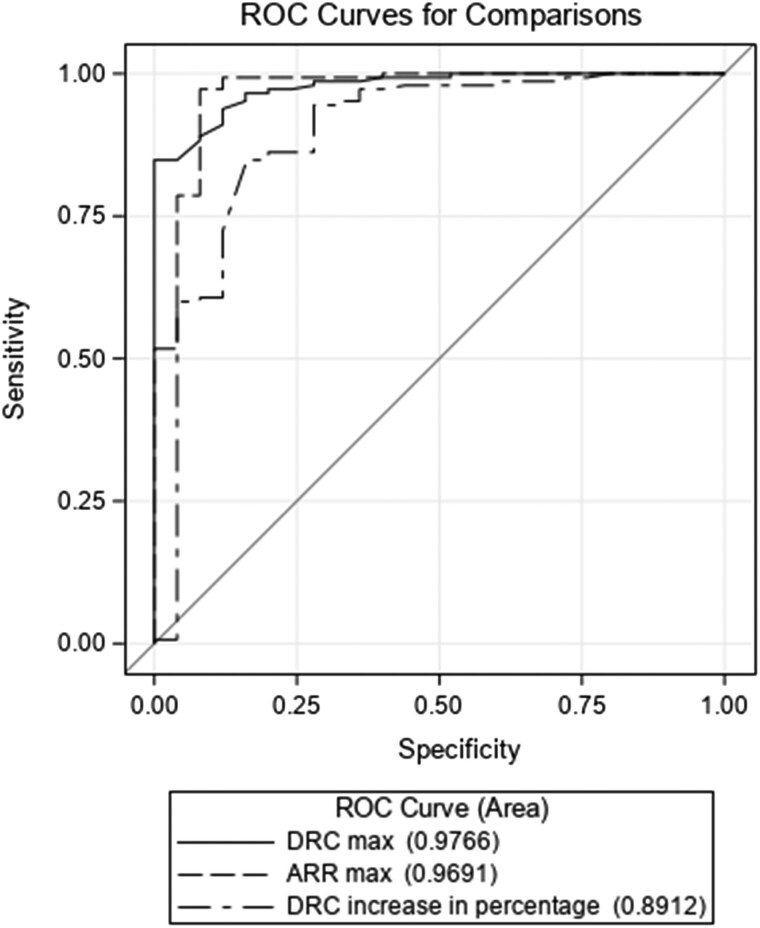
ROC curve analysis for the best predictors of primary aldosteronism during the upright posture stimulation test. Abbreviations: ROC, receiver operating characteristic.

During the UPT, from ROC curve analysis (Fig. 4) and best cut-point selection, a DRC_max_ ≤12 ng/L, a maximal ARR ≥ 58.5 (pmol/L/ng/L), and an increase of ≤108.3% in DRC (data not shown) were all found to be associated with a diagnosis of PA, without any statistical difference between the AUC of these 3 predictors (*P* > .05).

For convenience purposes, we chose to use the DRC_max_ for interpretation of the UPT results with an AUC (95% CI) of 0.98 (0.96; 1.00) and an odds ratio of 0.761 (both *P* < .0001). These results were concordant with our hypothesis that UPT can diagnose PA when aldosterone increases but renin remains suppressed. The sensitivity, specificity, and accuracy of DRC_max_ ≤12 ng/L for PA diagnosis were 93.8%, 88%, and 92.9%, respectively.

As shown in Table 3, 95.6% of patients with PA and a suppressed DRC_max_ (≤12 ng/L) increased PAC by at least 50% during the UPT (median increase of 222.2%), despite renin remaining suppressed. During the UPT, PAC and DRC both increased in all 42 non-PA patients, similarly to the response seen in the control group, with a median DRC_max_ of 23 (13, 52) ng/L (data not shown). This category of patients included 18 with secondary aldosteronism [median PAC_max_ 1183 (1047.25, 1463.25) pmol/L and median DRC_max_ 55 (28.0, 75.9) ng/L], 19 with a normal response to the UPT [median PAC_max_ 1015 (655.5, 1523.5) pmol/L and median DRC_max_ 18 (13.0, 25.1) ng/L], and 2 with uncertain results (PAC persistently < 277 pmol/L during the UPT and a suppressed DRC ≤12 ng/L), and 3 had a ≥ 50% increase in cortisol during the test, thereby making UPT results noninterpretable (data not shown).

**Table 3.** PAC increase (percentage intervals) during the UPT in patients diagnosed with PA who have a persistently suppressed DRC during the UPT (n = 136) and in patients diagnosed with PA who have a persistently suppressed DRC during the UPT along with available IV saline test results (n = 82)

PAC increase (% intervals)	Confirmed PA + DRC_max_ ≤12 ng/L (n = 136) (%)	Confirmed PA + DRC_max_ ≤12 ng/L + IV Saline test available (*n* = 82)			
		Total (*n* = 82)	Normal (*n* = 41)	Borderline (*n* = 24)	Elevated (*n* = 17)
[50-1162]	130 (95.6)	79 (96.3)	40 (97.6%)	24 (100%)	15 (88.2%)
[100-1162]	119 (87.5)	72 (87.8)	37 (90.2%)	21 (87.5%)	14 (82.4%)
[150-1162]	99 (72.8)	62 (75.6)	33 (80.5%)	17 (70.8%)	12 (70.6%)
[200-1162]	77 (56.6)	50 (61.0)	28 (68.3%)	13 (54.2%)	9 (52.9%)
[250-1162]	56 (41.2)	36 (43.9)	18 (43.9%)	10 (41.7%)	8 (47.1%)
[300-1162]	36 (26.5)	23 (28.0)	13 (31.7%)	5 (20.8%)	5 (29.4%)
[350-1162]	28 (20.6)	19 (23.2)	12 (29.3%)	3 (12.5%)	4 (23.5%)
[400-1162]	22 (16.2)	14 (17.1)	10 (24.4%)	1 (4.2%)	3 (17.6%)

Results are presented as n (%).

Abbreviations: DRC, direct renin concentration; IV, intravenous; PA, primary aldosteronism; PAC, plasma aldosterone concentration; UPT, upright posture stimulation test.

**Figure 5.**
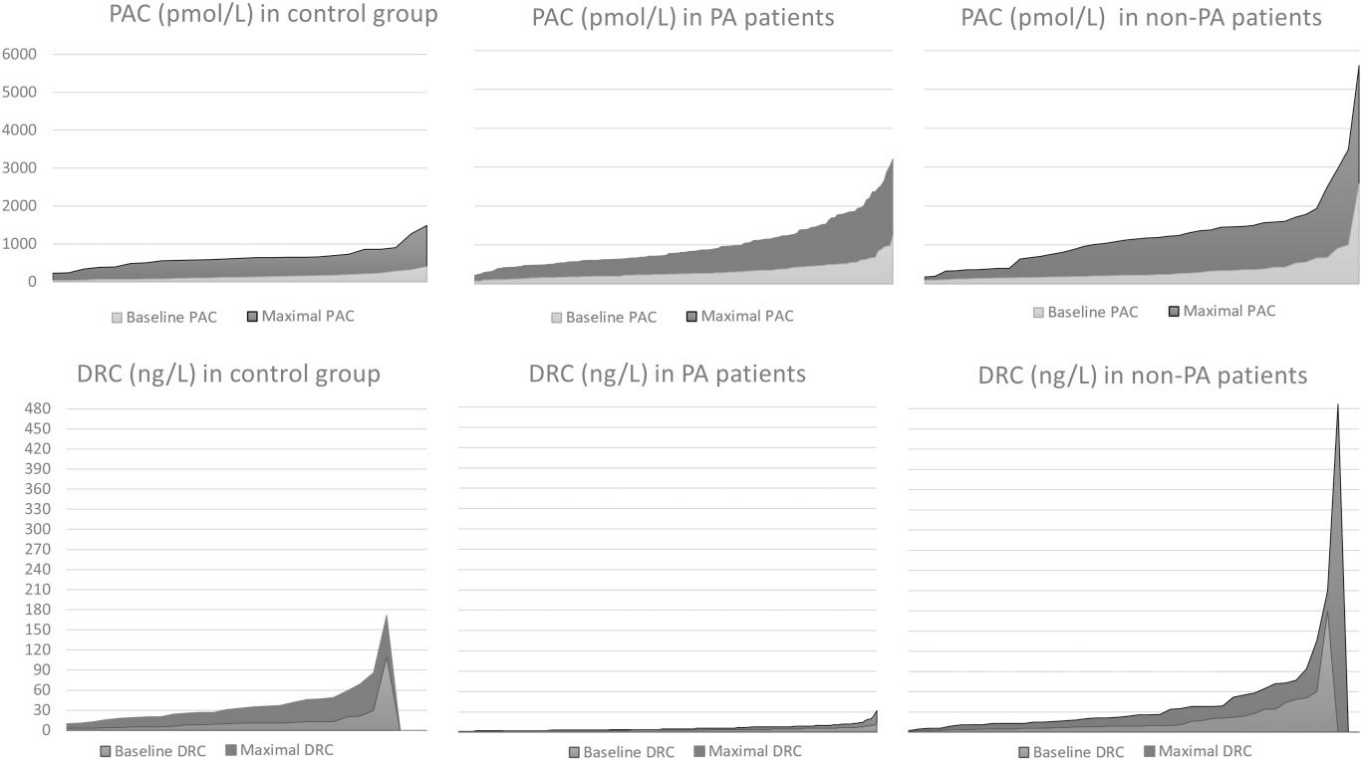
The variability in PAC (pmol/L) and in DRC (ng/L) at baseline and at maximal value following ambulation during the upright posture stimulation test in each of the 3 groups: the control group, the patients with primary aldosteronism (PA patients), and the hypertensive patients without primary aldosteronism (non-PA patients), respectively. Abbreviations: DRC, direct renin concentrations; PAC, plasma aldosterone concentrations.

Despite the differences in the response patterns seen for PAC and DRC in each of the controls, PA patients, and non-PA patients during the UPT, there was a wide variability in PAC and DRC values during the test (Fig. 5). As such, in PA patients, PAC varied at baseline from values less than a 100 to more than 1000 pmol/L (maximal 3300 pmol/L) following an upright posture, with a wide variability. Less variability was seen with DRC in response to the UPT in patients with confirmed PA: DRC remained suppressed (≤12 ng/L) in most patients and reached higher values in only a small number. The same observation can be made in controls and non-PA patients, where a wide variability was seen in their responses. However, control subjects had lower extremes of PAC, and non-PA patients had higher extremes of PAC; both achieved higher DRC (Fig. 5).

### UPT in PA Patients With False-Negative IV ST

A total of 89 PA patients (89/145) underwent both the IV ST and UPT. This subgroup was analyzed according to the results of the IV ST. Forty-one patients had a normal suppression (false-negative) (PAC <162 pmol/L) on IV ST: all of them had a DRC_max_ ≤12 ng/L during the UPT, and 97.6% increased PAC by at least 50%. Twenty-seven patients had a borderline suppression (PAC between 162 and 240 pmol/L) on IV ST: during UPT, 24 of them (88.9%) had a DRC_max_ ≤12 ng/L, and all increased PAC by at least 50%. Of the 21 patients with confirmed PA based on failed suppression of PAC during IV ST (≥240 pmol/L), 81% had a DRC_max_ ≤12 ng/L, and 88.2% increased PAC by at least 50% during UPT. Only the detailed results of the 82 patients (82/89) with a suppressed DRC_max_ during the UPT are shown in Table 3.

Without the UPT and based solely on IV ST results, only 23.6% of this selected group of patients (21/89) could unequivocally be diagnosed with PA (53.9%) if borderline results were also considered diagnostic, thus increasing the number of patients with a positive result on IV ST to 48. This increased to 88.8% when interpreting the UPT (79/89).

### UPT According to Lateralization on AVS

**Figure 6.**
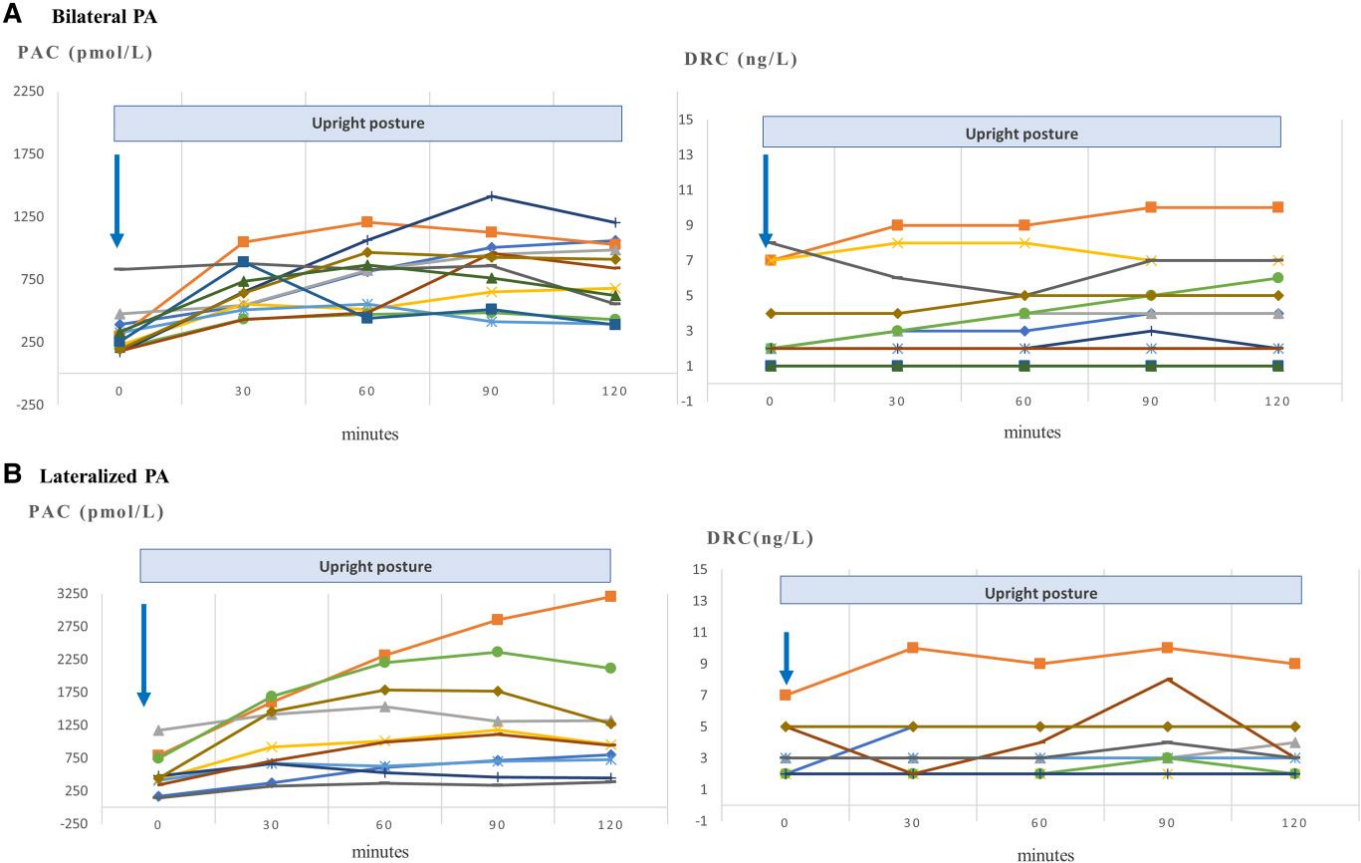
Comparison of the response of each of PAC (pmol/L) and DRC (ng/L) to the upright posture stimulation test, between (A) 11 patients with bilateral PA (11/35) and (B) 10 patients with lateralized PA (10/28) diagnosed based on adrenal vein sampling results. Abbreviations: DRC, direct renin concentrations; PA, primary aldosteronism; PAC, plasma aldosterone concentrations.

Finally, 63 patients underwent AVS for identification of the source of PA; 44.4% (n = 28) had lateralized PA, with higher baseline PAC, the only variable significantly different from bilateral PA (*P* = .043), reflecting a more severe PA (Table 4). However, the PAC and renin responses to the UPT did not significantly differ between the 2 subtypes of PA, as shown in Fig. 6, (illustrative selected number of patients from each group).

**Table 4.** Comparison of response to the upright posture stimulation test between patients with lateralized PA (n = 28) and patients with bilateral PA (n = 35), according to adrenal vein sampling results

	Lateralized PA (n = 28)	Bilateral PA (n = 35)	*P*-value*^a^*
	Median (IQR)	Median (IQR)	
PAC basal (pmol/L)	383.3 (245.5, 496.8)	265.5 (190.5, 366.5)	.**0434**
PAC max (pmol/L)	963.5 (700.5, 1487.0)	877.0 (569.0, 1206.0)	.1370
DRC basal (ng/L)	3.0 (2.0, 4.8)	3.0 (2.0, 5.0)	.9272
DRC max (ng/L)	4.0 (3.0, 7.0)	4.0 (2.5, 7.0)	.8507
C basal (nmol/L)	215.8 (149.3, 290.8)	269.5 (192.5, 321.5)	.0969
C max (nmol/L)	294.0 (218.0, 375.5)	319.0 (257.0, 390.0)	.2106
ARR basal (pmol/L/ng/L)	129.9 (64.3, 193.6)	89.3 (47.6, 165.7)	.1117
ARR max (pmol/L/ng/L)	318.4 (149.8, 478.3)	239.7 (125.8, 398.3)	.2931
PAC increase (%)	220.5 (138.3, 282.0)	205.6 (149.4, 291.6)	.6883
DRC increase (%)	50.0 (29.2, 85.7)	33.3 (0.0, 66.7)	.1743
Cortisol increase (%)	24.8 (3.9, 73.8)	8.0 (−7.7, 71.7)	.2806
ARR increase (%)	131.5 (67.6, 194.2)	151.9 (74.0, 248.2)	.3329
Potassium (mmol/L)	3.5 (3.2, 3.8)	3.8 (3.3, 4.0)	.0788

Bold *P*-value ≤.05 = statistically significant.

Abbreviations: ARR, aldosterone to renin ratio; C, cortisol concentration; DRC, direct renin concentration; IQR, interquartile range; Max, maximal; PA, primary aldosteronism; PAC, plasma aldosterone concentration.

^*a*^Kruskal–Wallis test was used for comparison.

### Postoperative Outcomes

Unilateral adrenalectomy was performed in 24 patients with PA. Two patients were operated in other institutions without available histopathology results and were not included in the following analysis. Of the 22 remaining patients, 20 had a lateralized aldosterone source on AVS and 2 had bilateral severe asymmetrical disease. In this subgroup, 20/22 patients had a UPT response consistent with PA (DRC_max_ ≤ 12); the remaining 2 had PA confirmed with a positive IV ST. Based on histopathology studies, 8/22 patients had confirmed adenoma and 3 had a single nodule, but surgery was done prior to CYP11B2 immunohistochemistry (IHC) availability; 10 had 1 dominant nodule and/or multiple adjacent micronodules (9/10 with positive CYP11B2 IHC); and 1 had adrenal nodular hyperplasia (no IHC) and had a positive oral saline test and a positive posture test. Eight patients with positive CYP11B2 IHC had a positive posture test, of which 1 had a negative IV ST, 2 had borderline IV ST, 1 had no test performed other than UPT, 2 had positive IV ST, and 2 had both positive IV and oral ST. Only 1 patient with positive CYP11B2 IHC had a negative UPT, while PA was confirmed by IV ST (borderline result).

Postoperative clinical and biochemical follow-up data was available for 17 patients. The median (interquartile range) time of follow-up after surgery was 24 (13, 36) months. Potassium was normalized in 16 patients (1 had missing data). Nine patients had ARR done at follow-up: all were normal [median 13.22 (11.25, 24.19) pmol/L/ng/L (cut-off of 144 according to the 2016 Endocrine Society clinical practice guideline)]. Blood pressure at follow-up was available for 15 patients and 11 had normal blood pressure (below 140/90). The number of antihypertensive medication postoperatively was reduced in 5 patients and medication was completely stopped in 6 patients.

## Discussion

The primary objective of this study was to examine whether the UPT can be useful in the confirmation of PA diagnosis, particularly in a selected group of patients with borderline or potentially false-negative ST. We demonstrate that in 89.6% of such selected patients (130/145), the UPT induced a renin-independent increase in PAC of at least 50%, supporting PA diagnosis. The response to an upright postural stimulation in patients with PA is inherently different from that seen in normotensive control subjects whose renin-angiotensin aldosterone axis (RAA) responded physiologically [34], with proportional increases of both renin and PAC (Spearman correlation = 0.44). In the early studies on the RAA axis, Cohen et al demonstrated an increase in PRA in normal subjects as early as 15 minutes after the start of ambulation, with a peak at 60 or 120 minutes (from a mean of 166 ± 35 ng/dL at baseline to a mean of 477 + 97 ng/dL at 60 minutes, *P* = .025, and 543 ± 87 ng/dL at 120 minutes, *P* < .005) and a parallel increase in urinary aldosterone excretion (from a mean of 0.7 ± 0.1 µg/hour at baseline to a mean of 1.97 ± 2.7 µg/hour at 120 minutes, *P* < .005) [34].

In contrast, in PA patients with sufficient aldosterone excess, the RAA axis is suppressed and renin cannot be stimulated normally, even by physiological stimuli such as salt restriction or diuretics (volume contraction) or posture [40, 41]. In this study, we found that in 95.6% of patients with PA and suppressed DRC, PAC increases by at least 50% in response to an upright posture. A suppressed renin of ≤12 ng/L was found to successfully predict PA during the UPT, with an AUC (95% CI) of 0.98 (0.96; 1.00) and an accuracy of 92.9%. The optimal cut-off for PAC increase allowing the best sensitivity to specificity ratio was 373.9% (data not shown), while the median PAC increase in our study population was 222%; however, it varied widely from a minimum of 24.3% to a maximum of 1162% (data not shown) in PA patients. Several studies have previously demonstrated the variability of aldosterone concentrations in PA, even for intraindividual measurements, thus a limiting factor in setting fixed diagnostic thresholds for PA [9, 10, 13, 42]. As many as 29% of our patients had at least 1 aldosterone measurement below conventional diagnostic thresholds for PA (ie, <277 pmol/L), and 38% had an ARR below 70 pmol/L/mU/L [10, 42]. This is in line with studies showing the important effect of posture on aldosterone in PA and that using a cut-off of 277 pmol/L for PA screening could miss 14.3% of patients with true PA [4, 13, 43]. Several studies also reported unusually low aldosterone concentrations in patients undergoing AVS, as they are in a supine posture [43-45]. In our study, we would have missed as much as 50% of PA patients if we had only considered the baseline supine aldosterone levels. Most of our PA patients increased their PAC by more than 50% from baseline, but those who did not could still be diagnosed based on a persistently suppressed DRC. Thus, no efficient cut-off for PAC increase during the UPT could unequivocally predict PA, which is in line with previous observations regarding aldosterone inherent variability in PA, as discussed earlier, and a consistently suppressed renin provides an increasingly added value for PA diagnosis.

In our centers, the seated IV ST was part of our usual initial confirmation test for PA in patients with suggestive clinical and biochemical screening. We did, however, observe that a nonnegligible number of cases had possible false negative results to the seated IV ST, and we wished to explore an alternative diagnostic approach. Stowasser et al previously found that during seated IV ST, PAC can be suppressed to below 162 pmol/L (false negative) in 10.4% of patients otherwise proven to have PA; this false-negative rate increased to 54.5% when the IV ST was performed in a recumbent posture [33]. This further supports the influence posture exerts on aldosterone concentrations leading to a more pronounced response to the IV ST. During our previous studies that examined the potential role of aberrant expression of several GPCR in PA adrenal tissue, we found that several stimuli could increase aldosterone secretion in a renin-independent manner. In a group of 43 patients with PA, a renin-independent aldosterone increase of ≥50% from baseline during the 2-hour UPT was observed in 83% and 71% of patients with BAH and APA, respectively [29]. The UPT in this prospective study was performed following dexamethasone suppression for 3 days, just as other aberrant GPCR screening tests are usually performed, to eliminate the effect of endogenous ACTH on aldosterone production [29, 30]. In contrast, the UPT in the present study was not performed with dexamethasone suppression, as this usually decreases aldosterone secretion by approximately 50% in PA patients [46]. However, we did stipulate that cortisol should not increase above 50% during upright stimulation to correctly interpret results as independent of ACTH effects.

**Figure 7.**
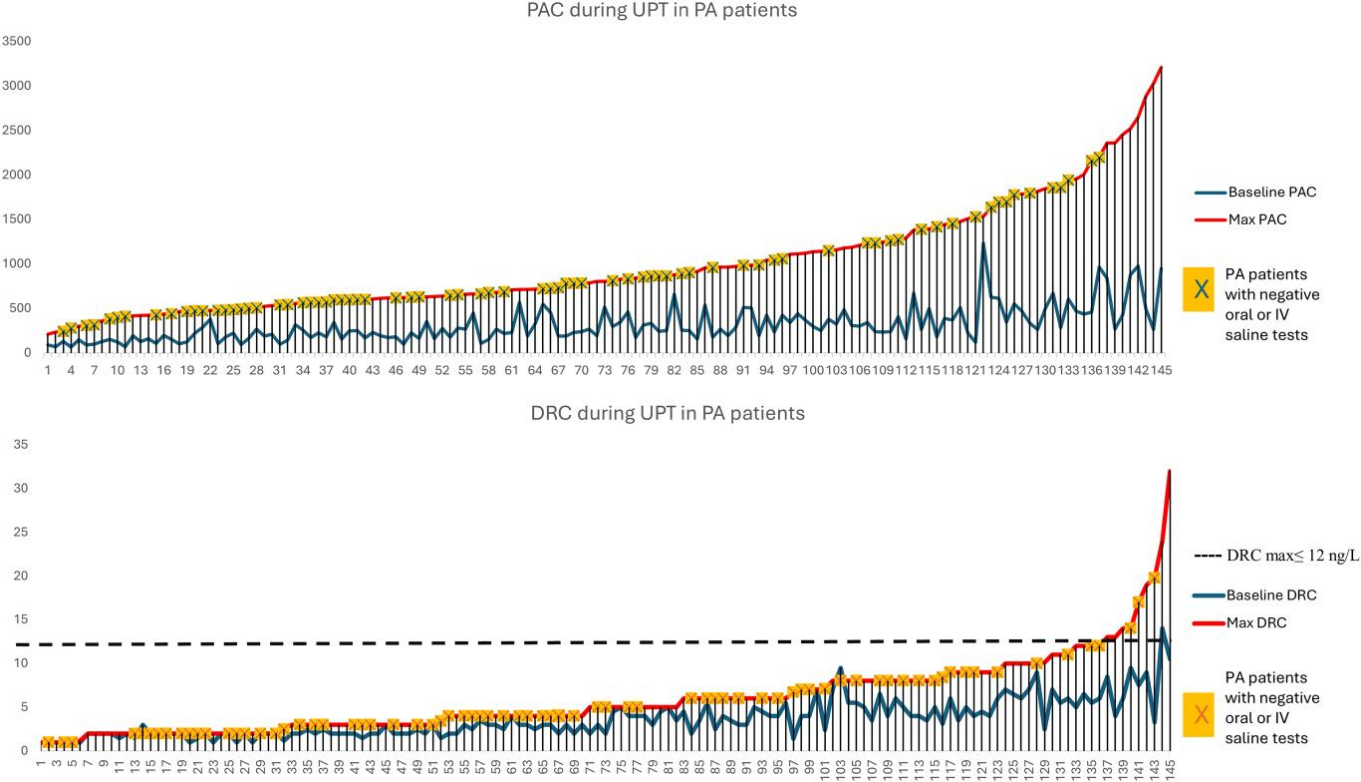
PAC and DRC during the UPT in patients with PA highlighting the individuals (with an X) who had a negative oral or intravenous saline test result. Abbreviations: DRC, direct renin concentrations; PA, primary aldosteronism; PAC, plasma aldosterone concentrations; UPT, upright posture test.

When we compared IV ST and UPT results in the subgroup of PA patients who underwent both tests, we found that the UPT was most useful in patients with a high clinical and biochemical suspicion of PA and either a negative or a borderline result on the IV ST, who would otherwise have been ruled-out as non-PA. In fact, in these patients, the role of a suppressed renin in diagnosing PA is further emphasized, especially when coupled with an increase in PAC of at least 50% (Fig. 7). If we had only considered the ST results (both normal and borderline), we would have misclassified approximately 64 patients as having essential hypertension (Table 3). This is further corroborated by Parksook et al who reported that 15% of patients with negative IV ST in the low-renin hypertension population had a nonsuppressible aldosterone production and lateralized on AVS, thus making SST unable to correctly diagnose a significant number of patients with PA who would otherwise benefit from PA-targeted therapies [47].

Whereas the UPT was used in previous studies in an attempt to distinguish PA etiologies between APA and BAH, we found no difference in responses between the 2 etiologies. Previously, in patients with an APA, PAC was expected to either decrease or increase to less than 30% from baseline after 4 hours of ambulation [22, 27, 48]. Also, variations were expected in response to the diurnal rhythm, as ACTH would be responsible for higher levels in the early morning, and this could be partially abolished by the administration of dexamethasone [26]. However, these results are not always reproducible, as among patients with APA, response to the UPT was heterogeneous and some were found to have an increment in PAC after ambulation [19, 29, 49, 50]. In patients with BAH, whose aldosterone excess was less severe, it was believed that the incomplete suppression of the RAA axis could explain the barely detectable increase in renin and angiotensin-II and the increase of PAC following ambulation. However, these hypotheses were never confirmed; therefore, the UPT provided little to no usefulness and was mostly abandoned for differentiating between PA etiologies. Indeed, in 2 prospective studies, the sensitivity of the UPT was as low as 44% to 64% for PA subtyping [28, 49]. Furthermore, our present study, along with others, demonstrated a renin-independent PAC increase in a high proportion of patients with APA or BAH in response to posture [29, 49, 51, 52].

Although the number of our patients undergoing surgery is relatively limited, PA diagnosis was confirmed in 9 patients with positive immunostaining for CYP11B2 following adrenalectomy based upon positive UPT in 8 patients and, interestingly, in 4/8 patients with no other positive tests (negative or borderline ST or no other tests performed); pathology findings in other patients operated prior to CYP11B2 IHC were also supportive of PA diagnosis. This further supports the usefulness of UPT as a potential confirmatory test of PA in patients with clinical suspicion but with uncertain response to saline suppression tests. In addition, our postoperative clinical and biochemical follow-up data, while limited, provided similar outcome results as in other studies using the PASO criteria [37].

We found mild cortisol cosecretion, defined by a morning cortisol >50 nmol/L following late night 1 mg-DST, in 23.6% of PA patients who had undergone the test. The prevalence of cortisol cosecretion ranges from 5% to 77% in the literature; this wide variation translates to differences in the adopted definition of cortisol cosecretion between studies [53-56].

The French Society of Endocrinology consensus on PA subtyping recommends against postural stimulation testing [57], while the Endocrine Society guidelines [8] reserve it for specific cases where AVS was unsuccessful. None of the guidelines include the UPT with the initial confirmatory tests, with the exception of the Japan Endocrine Society clinical practice guideline [15], which include a variation of the UPT, the furosemide-upright posture test discussed earlier. We also suggest using the UPT for PA confirmation rather than subtyping. We defined the following optimal cut-off point for maximal DRC achieved during the UPT: ≤ 12 ng/L, allowing for easy interpretation of the test while maintaining the best combination of sensitivity and specificity.

The underlying mechanisms explaining the response of aldosterone to the UPT are yet to be elucidated. It was initially thought that because BAH retained responsiveness to angiotensin II, aldosterone increased when standing [58, 59]. Subsequently, as much as 60% of APA was also found to maintain angiotensin II responsiveness, allowing for increasing confusion when interpreting the UPT [25, 51]. Nonetheless, a Japanese study including patients with APA demonstrated that aldosterone increased in response to postural stimulation but infusion of angiotensin II failed to reproduce the same results, thereby challenging the role attributed to angiotensin II in the response to posture [19]. In normal subjects, a series of neurohormonal events occur following standing in response to the decrease in plasma volume and in the systolic blood pressure. An increase in epinephrine, norepinephrine, PRA, aldosterone, and vasopressin is seen [60]. In PA patients, however, the response of aldosterone to the upright posture, which is independent from renin and partly from ACTH, could be driven by the stimulation of aberrant G-protein coupled receptors expressed in adrenal tissue, such as the beta-adrenergic or the vasopressin receptor, especially because the aldosterone response is more significant when beta-blockers and angiotensin receptor antagonists are withdrawn before performing the UPT [29, 32]. Furthermore, another receptor, not yet identified, could potentially be responsible for the response seen following ambulation in PA. Prospective in vivo and in vitro studies are needed to further explain the underlying mechanisms governing the UPT responsiveness in PA.

Our study has several limitations. First, we did not prospectively compare the UPT to a gold-standard confirmatory test for PA, such as the fludrocortisone suppression test, because it is a cumbersome test, or to other commonly used confirmatory tests because of their significant false-negative rate and the risk of misdiagnosing true PA as non-PA. Second, some of our patients had PRA measurements while others had DRC measurements, which are qualitatively different assays. We used the following conversion factor to simplify our analysis: 1 ng/mL/hour = 10 ng/L, to convert from PRA to DRC, despite poor correlation between both measurements, particularly when PRA is suppressed to below <1 ng/mL/hour, which is the case for most PA patients [8]. However, only 17% of our total population had PRA measurements, of which 41.6% were in the control group and had nonsuppressed PRA, limiting the influence the type of assay has on our analysis. Also, we used the cut-off suggested by Stowasser et al for the interpretation of the seated IV ST, despite using a different radioimmunoassay for plasma aldosterone measurement in our study [33]. Reported PAC with this method are usually higher compared to liquid chromatography-tandem mass spectrometry due to a lower specificity, and thus a higher cut-off may have been more appropriate, such as 183 pmol/L, as suggested by a relatively newer study on seated ST when radioimmunoassay is used [61]. However, because most of our patients had a very high clinical suspicion of PA and a high pretest probability, we preferred a stricter cut-off to reduce the risk of false negatives. Another limitation is the fact that different assays were used for aldosterone and renin measurements throughout our inclusion period, but we believe that our results remain pertinent since we were mostly using ratios rather than absolute values but recognize that the cut-off identified for renin during UPT could be different with current more precise assays. Finally, we mostly performed the UPT in patients with borderline results on the ST. Larger studies encompassing all consecutive cases including milder forms of PA (especially because this category of patients has been shown to be the most difficult to diagnose) should be performed to better characterize the usefulness of UPT in the confirmation of PA [9]. Further studies are also needed to see whether stopping interfering antihypertensive drugs 3 days prior to testing is sufficient to unmask PAC's response to the UPT and evaluate the need for dexamethasone suppression before testing to eliminate the effect of ACTH on aldosterone. In addition, future prospective studies using more precise assays and looking at treatment outcomes (both surgical and medical) in patients with PA diagnosed on the basis of UPT should be conducted to validate the accuracy of this test and its role in PA diagnosis.

In conclusion, we found that UPT can be useful for PA confirmation, especially in patients with a high clinical or biochemical suspicion and negative/borderline ST results, potentially explained by aldosterone's demonstrated intraindividual variability. Among patients with PA, a high proportion responds to posture stimulation by increasing aldosterone secretion in a renin-independent manner, which was not seen in normotensive subjects and hypertensive patients without PA. There was no difference in the response pattern to upright stimulation between lateralized and bilateral PA. We suggest that the usefulness of UPT in the diagnostic approach for PA confirmation rather than PA subtyping should be explored further in prospective studies with a combined exploration of its biological and molecular determinants.

## Data Availability

Some or all datasets generated during and/or analyzed during the current study are not publicly available but are available from the corresponding author on reasonable request.
